# Supplementary material for: Intraocular Pressure-Lowering and Retina-Protective Effects of Exosome-Rich Conditioned Media from Human Amniotic Membrane Stem Cells in a Rat Model of Glaucoma
Source: Int J Mol Sci. 2023 Apr 29;24(9):8073. doi: 10.3390/ijms24098073 (PMC10179312; doi:10.3390/ijms24098073)
Supplement: Supplementary file 1 [file ijms-24-08073-s001.zip › ijms-2341118-supplementary.pdf]

International Journal of *Molecular Sciences*

MDPI

Manuscript ijms-2341118

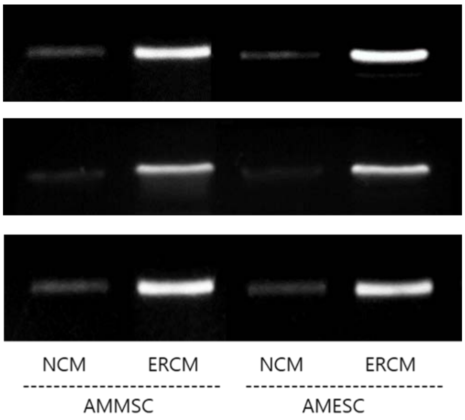

Supplementary Figure S1. Western blot analysis of CD9-positive exosomes.

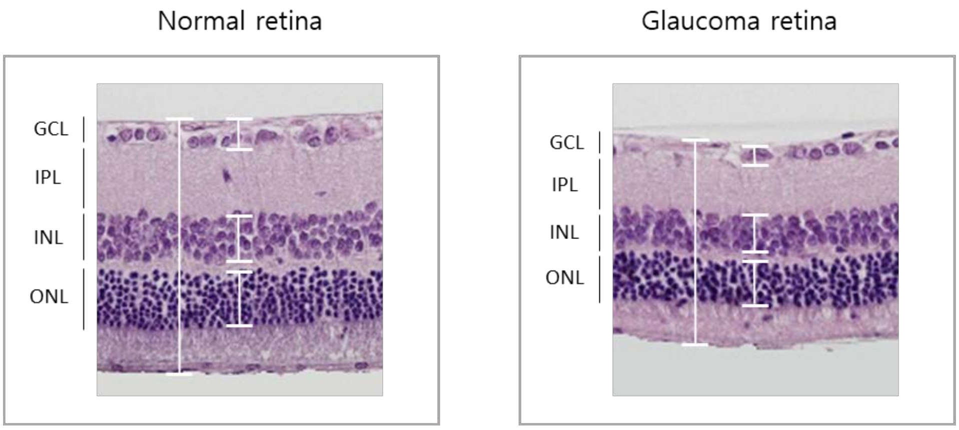

Supplementary Figure S2. Retinal structure of normal and glaucoma rats. GCL: ganglion cell layer, IPL: inner plexiform layer, INL, inner nuclear layer, ONL: outer nuclear layer.

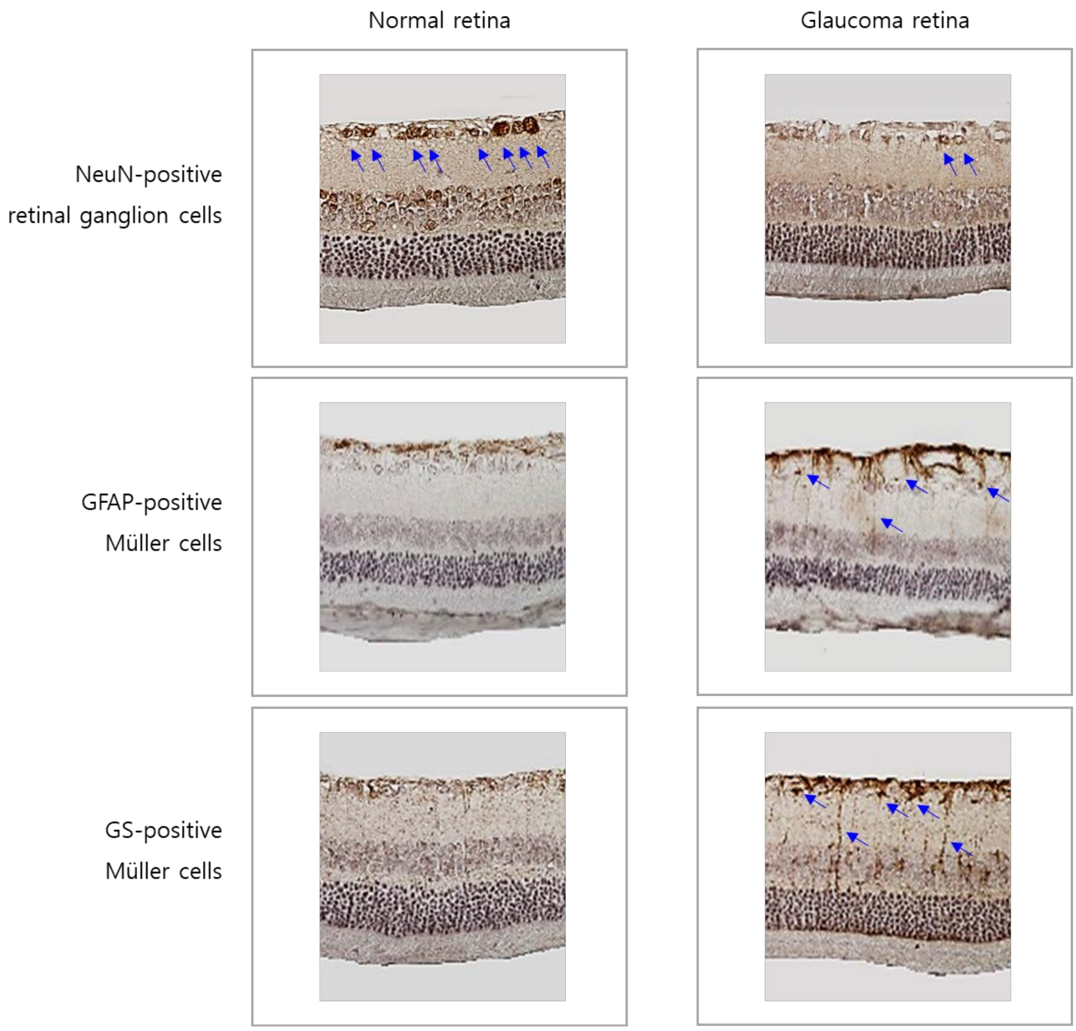

Supplementary Figure S3. Immunohistochemical findings of NeuN-positive retinal ganglion cells (A, arrow), glial fibrillary acidic protein (GFAP)-positive Müller cells (B, arrow), and glutamine synthetase (GS)-positive Müller cells (C, arrow) in the retina of normal and glaucoma rats.
